# Reproductive Status Is Associated with the Severity of Fibrosis in Women with Hepatitis C

**DOI:** 10.1371/journal.pone.0044624

**Published:** 2012-09-10

**Authors:** Erica Villa, Ranka Vukotic, Calogero Cammà, Salvatore Petta, Alfredo Di Leo, Stefano Gitto, Elena Turola, Aimilia Karampatou, Luisa Losi, Veronica Bernabucci, Annamaria Cenci, Simonetta Tagliavini, Enrica Baraldi, Nicola De Maria, Roberta Gelmini, Elena Bertolini, Maria Rendina, Antonio Francavilla

**Affiliations:** 1 Department of Gastroenterology, Azienda Ospedaliero-Universitaria & University of Modena and Reggio Emilia, Modena, Italy; 2 Sezione di Gastroenterologia, Di.Bi.M.I.S., University of Palermo, Palermo, Italy; 3 Department of Gastroenterology, University of Bari, Bari, Italy; 4 Department of Pathology, Azienda Ospedaliero-Universitaria, Modena, Italy; 5 Department of Clinical Pathology, NOCSAE, Modena, Italy; 6 Department of General Surgery, Azienda Ospedaliero-Universitaria, Modena, Italy; 7 Istituto di Ricovero e Cura “Saverio de Bellis”, Castellana Grotte, Italy; University of Montreal, Canada

## Abstract

**Introduction:**

Chronic hepatitis C is the main cause of death in patients with end-stage liver disease. Prognosis depends on the increase of fibrosis, whose progression is twice as rapid in men as in women. Aim of the study was to evaluate the effects of reproductive stage on fibrosis severity in women and to compare these findings with age-matched men.

**Materials and Methods:**

A retrospective study of 710 consecutive patients with biopsy-proven chronic hepatitis C was conducted, using data from a clinical database of two tertiary Italian care centers. Four age-matched groups of men served as controls. Data about demographics, biochemistry, liver biopsy and ultrasonography were analyzed. Contributing factors were assessed by multivariate logistic regression analysis.

**Results:**

Liver fibrosis was more advanced in the early menopausal than in the fully reproductive (P<0.0001) or premenopausal (P = 0.042) group. Late menopausal women had higher liver fibrosis compared with the other groups (fully reproductive, P<0.0001; premenopausal, P = <0.0001; early menopausal, P = 0.052). Multivariate analyses showed that male sex was independently associated with more severe fibrosis in the groups corresponding to premenopausal (P = 0.048) and early menopausal (P = 0.004) but not late menopausal pairs. In women, estradiol/testosterone ratio decreased markedly in early (vs. reproductive age: P = 0.002 and vs. premenopausal: P<0.0001) and late menopause (vs. reproductive age: P = 0.001; vs. premenopausal: P<0.0001). In men age-matched with menopausal women, estradiol/testosterone ratio instead increased (reproductive age group vs. early: P = 0.002 and vs. late M: P = 0.001).

**Conclusions:**

The severity of fibrosis in women worsens in parallel with increasing estrogen deprivation and estradiol/testosterone ratio decrease. Our data provide evidence why fibrosis progression is discontinuous in women and more linear and severe in men, in whom aging-associated estradiol/testosterone ratio increase occurs too late to noticeably influence the inflammatory process leading to fibrosis.

## Introduction

Chronic hepatitis C (CHC) is the primary cause of death in patients with end-stage liver disease [1]. Its prognosis depends on the accumulation of fibrosis over time due to various mechanisms of tissue damage caused by viral infection, ultimately leading to the development of cirrhosis and related complications. The development of hepatic fibrosis is most directly correlated with necro-inflammation, and several other host and viral factors have been associated with the rate of fibrosis progression [2], including older age, alcohol consumption, duration of infection, viral co-infections, steatosis, insulin resistance, and vitamin D deficiency [3].

Studies of large cohorts of patients with CHC have also found that high levels of estrogens (as observed during pregnancy) [4] are associated with decreased inflammatory activity in HCV women and that the progression of fibrosis in CHC is twice as rapid in men as in women [5], [6]. This difference has been attributed to the protective role of estrogens and has been supported by experimental and clinical data. Experimentally, estrogens were shown to have a relevant fibrosuppressive role in a rat model of dimethylnitrosamine- or pig-serum-induced liver fibrosis [7], [8]. Clinically, Di Martino et al. [9] and Codes et al. [10] showed that the progression of fibrosis increased significantly in women after menopause, whereas prolonged periods of hormone replacement therapy (HRT) were able to maintain this progression at a level similar to that in premenopausal women. In women, menarche initiates a long period of estrogen exposure that begins to decline in the premenopausal period, often leading to undetectable estrogen levels in early and late menopause. In women with CHC, this reduction in estrogen levels is accompanied by consensual fluctuation in the levels of pro-inflammatory [11], [12] and anti-inflammatory [12] cytokines that could interfere with the course of necro-inflammation and the progression of fibrosis. Much less is known about this process in men: pro-inflammatory cytokines do not fluctuate in age subgroups corresponding to female reproductive stages [11], but it remains unknown whether a relationship exists with changes in sex hormone balance in men.

Thus, the aim of our study was to evaluate the effects of differential hormonal exposure on the severity of fibrosis in men and women: we therefore compared women with CHC in different reproductive phases (reproductive age, premenopause, early menopause, late menopause) with four age-matched groups of men and investigated the correlations between fibrosis and the respective Estradiol and Testostosterone levels.

## Materials and Methods

Between January 2002 and December 2008, 1000 consecutive patients with CHC were recruited to receive standard antiviral treatment at the Gastrointestinal and Liver Units of the University Hospitals of Modena and Bari, Italy. Eligible patients were ≥18 years of age, had compensated liver disease due to chronic hepatitis C virus (HCV) infection (any fibrosis stage, including compensated cirrhosis), detectable plasma HCV RNA levels, and had received no previous treatment for hepatitis C. Patients were excluded if they were co-infected with human immunodeficiency virus or hepatitis B virus or had any other cause of liver disease, severe depression or psychiatric disorder, or active substance or alcohol consumption >20 g/day in the last five years, as evaluated by a questionnaire. Women who had been using hormone replacement therapy were not included in the analysis. The detailed demographic characteristics of this cohort of patients have been reported previously [11].

Within this cohort, four groups of women were selected according to reproductive stage:

Group 1: full reproductive age (i.e., regular menses); Group 2: premenopausal; women included in this group entered menopause (defined as no menstrual period for 12 consecutive months) within 5 years from enrollment in the study; Group 3: early menopausal (menopause was present at the time of enrollment for less than 5 years); Group 4: late menopausal (menopause was present at the time of enrollment for at least 10 years).

Four groups of men contained patients that were pair-matched by age (1∶1) with the female cohort.

This study was approved by the institutional review boards of the two hospitals and was conducted in accordance with the provisions of the Declaration of Helsinki and Good Clinical Practice guidelines (ClinicalTrials.gov identifier: NCT01402583).

### Clinical and Laboratory Assessment

All patients had undergone liver biopsy within 1 year before enrollment. Portal vein diameter (mm) was determined by color Doppler ultrasonography before liver biopsy. The following data were collected at the time of liver biopsy: age, sex, weight, height, and body mass index (BMI); serum levels of alanine aminotransferase (ALT), γ-glutamyl transpeptidase (GGT), glucose insulin; and platelet count. Insulin resistance was determined using the homeostasis model assessment (HOMA). HCV RNA was quantified by Abbott RealTime HCV assay (Abbott Molecular Inc., Des Plaines, IL, USA) and genotyped by INNO-LiPA assay (Innogenetics, Gent, Belgium). All biopsy specimens were reviewed and scored according to Ishak et al. [13] by a single pathologist (LL) who was blinded to the patients’ identities and histories. The percentage of hepatocytes containing macrovesicular fat was determined for each 10× field, and steatosis was classified as absent, mild (<5%), moderate (5–20%), or severe (≥20%).

### Hormone Assays

#### Estradiol and testostosterone

Blood samples were obtained from all patients by venipuncture and processed within 2 h after withdrawal. Serum was stored at –20°C and assayed to determine estradiol and testosterone levels. Serum samples were subjected to chemiluminescent microparticle immunoassays (CMIA) to determine 17β-estradiol and testosterone levels using commercially available kits and a c4000 Architect system (Abbott Diagnostic Division, Abbott Laboratories, Abbott Park, IL, USA). Estradiol was additionally quantitated using the Abbott Architect Estradiol Assay 200 (rev. 2004) one-step CMIA. Assay sensitivity was <10 pg/mL for estradiol and 50 pg/mL for testosterone. The intra- and inter-assay coefficients of variation were 5.6% and 4.9% for estradiol, and 4% and 4.6% for testosterone.

#### Anti-Müllerian hormone

Serum anti-Müllerian hormone (AMH) levels were assessed in female patients by enzyme-linked immunosorbent assay (AMH Gen II ELISA; Beckman Coulter, Inc., Brea, CA, US). The sensitivity of the assay was 0.08 ng/mL. Intra- and interassay coefficients of variation were <5% and <7%, respectively.

### Statistical Analysis

Continuous variables are summarized as mean (SD) and categorical variables as frequency (%). Continuous variables within each group were compared by the nonparametric Mann–Whitney U-test. Categorical data were compared by the chi-square test. Multiple logistic regression models were used to assess the relationship between severe fibrosis (Ishak staging ≥3) and the demographic, metabolic, and histological characteristics of patients in the individual groups and in each male–female pair group. In the statistical models, dependent variables were coded as 1 (present) or 0 (absent).

Regression analyses were performed using PROC LOGISTIC, PROC REG, and subroutines in SAS (SAS Institute, Inc., Cary, NC) [14].

## Results

The detailed epidemiological, virological, laboratorial, histological, and ultrasonographic results of each male–female pair group are presented in Tables S1 and S2. Women in each group were selected according to reproductive status (full reproductive age, premenopausal, early menopausal, late menopausal), and data from the female groups were compared with those from age-matched male groups (intragroup analysis, Mann-Whitney U test).

### Characteristics of Reproductive Status–Stratified Female Groups

We identified 123 women of full reproductive age [mean (SD), 36.7 (6.9) years; range, 18–45 years], 38 premenopausal women [mean (SD), 47.6 (1.8) years; range, 46–50 years], 50 women in the early menopausal stage [mean (SD), 53.8 (3.6) years; range, 47–60 years], and 144 women of late menopausal stage [mean (SD), 62.3 (3.2) years; range, 55–73 years].

**Figure 1:**
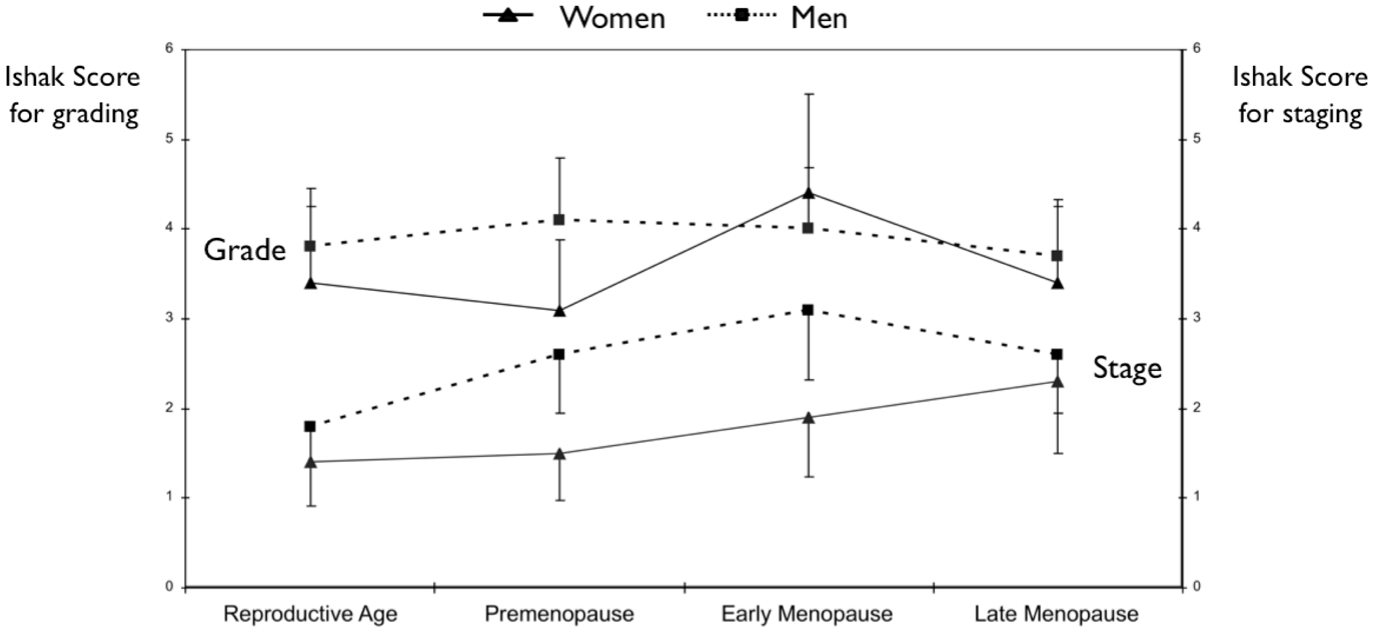
Mean necro-inflammation and fibrosis scores in the four subgroups of female and age-matched male patients with chronic hepatitis (Women: triangles; Men: squares). Levels of significance of the intra- and inter-group comparison are reported in the text.

The detailed comparison between the various groups is reported in Tables S3 and S4. Women in the first two cohorts (full reproductive age, premenopausal) showed similar moderately severe disease states, as indicated by liver necro-inflammatory activity (P = 0.42; Table S3) and staging (P = 0.062; Table S3, Figure 1). No significant difference in mean BMI was observed between these two groups (P = 0.118). Severe steatosis was more prevalent in premenopausal women (3/38, 8.0%) than in women of full reproductive age (4/123, 3.2%) although the difference did not reach significance (Table S4). The presence of cirrhosis in both groups at the time of enrollment was negligible.

The comparison of reproductive-aged women (full reproductive age, premenopausal) with those in early menopause revealed significant differences in several variables. The estimated duration of HCV infection was significantly longer in early menopausal women than in fully reproductive (P<0.0001) or premenopausal (P = 0.017) women. All indicators of disease severity were significantly worse in early menopausal women in comparison with the two reproductive-aged groups (early menopause vs. full reproductive age: grading, P = 0.020; staging, P<0.0001; early menopause vs. premenopause: grading, P = 0.014; staging, P = 0.042; Figure 1, Table S3). BMI did differ significantly among women in early menopause vs. full reproductive age (P = 0.039) but not between early menopause vs. premenopausal women (P = 0.90). A significantly higher percentage of early menopausal women had cirrhosis compared with reproductive-aged women (P = 0.039, Table S3).

In the late menopausal group, grading did not differ significantly in comparison with the reproductive-aged and premenopausal groups, but was significantly lower than that in the early menopausal group (P = 0.004). Staging at the time of liver biopsy was significantly higher in late menopausal women than in full reproductive age and premenopausal groups (P<0.0001 for both) while it was of borderline significance vs. early menopause (P = 0.052) (Table S3, Figure 1). Accordingly, cirrhosis was more prevalent in late menopausal women than in fully reproductive (P = 0.004) or premenopausal (P = 0.026) women; however, prevalence was not significantly different between late and early menopausal women (P = 0.25; Table S3). BMI, insulin resistance, and presence of steatosis did not differ significantly between any pair of female groups.

Multivariate analysis showed that necro-inflammation (OR: 1.506; 95% confidence interval (CI), 1.181–1.922, P = 0.001), steatosis (0 *vs*. >20%) (OR: 3.029; CI 1.154–7.951, P = 0.024), circulating estradiol levels (OR: 0.973, CI 0.947–0.999), P = 0.041), ALT (OR: 1.011, CI 0.003–1.019, P = 0.009) and portal vein diameter (OR: 2.644, CI 1.657–4.220, P<0.0001) were independently associated with the severity of fibrosis (Table 1). Neither age nor estimated length of HCV infection or estimated age of acquisition of infection was significantly related with severity of fibrosis.

**Table 1:** Univariate and multivariate analysis for fibrosis in the women with chronic hepatitis C.

	Univariate		Multivariate	
	OR (95% CI)	*P*	OR (95% CI)	*P*
**Age (years)**	1.089 (1.040–1.140)	0.000	1.028 (0.939–1.126)	0.553
**Duration of HCV infection (years)**	1.118 (1.037–1.205)	0.004	1.015 (0.884–1.166)	0.833
**HCV infection’s Acquisition Age**	1.036 (1.015–1.057)	0.001	1.000 (0.879–1.139)	0.996
**Necro-inflammation**	1.458 (1.277–1.665)	0.000	1.506 (1.181–1.1.922)	0.001
**Steatosis (0 ** ***vs*** **. >20%)**	1.775 (1.018–3.095)	0.043	3.029 (1.154–7.951)	0.024
**Circulating Estradiol (pgml)**	0.977 (0.963–0.991)	0.001	0.973 (0.947–0.999)	0.041
**Baseline HCV RNA (IU/mL)**	1.000 (1.000–1.000)	0.024	1.000 (1.000–1.000)	0.578
**ALT (IU/L)**	1.009 (1.004–1.013)	0.000	1.011 (0.003–1.019)	0.009
**GGT (IU/L)**	1.028 (1.016–1039)	0.000	1.008 (0.992–1.025)	0.327
**Platelet count (×10^3^/mm^3^)**	0.970 (0.969–0.981)	0.000	0.988 (0.974–1.002)	0.099
**Portal vein diameter (mm)**	2.392 (1.804–3.171)	0.000	2.644 (1.657–4.220)	0.0001

Only factors found to be significant by univariate analysis are reported.

### Comparison of Reproductive Status–Stratified Female and Male Groups

The results of the comparison of each male–female group pair are reported in Table S1. The estimated duration of HCV infection did not differ significantly between each male–female group pair. Men in groups paired with the first three female groups (reproductive age, premenopause, early menopause) showed remarkably more severe liver disease, as indicated by mean stage of fibrosis (P<0.0001 for all 3 groups; Table S1 and Figure 1), but no significant difference in fibrosis was found between women in the late menopausal group and age-matched men. Similarly, no significant difference in the presence of cirrhosis was observed between male–female pairs correlating with full reproductive age and late menopause, whereas the incidence of cirrhosis was significantly lower in the two intermediate groups of women than in age-matched groups of men (P = 0.003 for both; Table S1). In contrast, no difference in grading or steatosis was observed between male–female group pairs.

GGT levels were significantly lower in women than in men in all group pairs (full reproductive age, P<0.0001; premenopause, P = 0.001; early menopause, P = 0.007; late menopause, P = 0.017; Table S1). BMI differed significantly between men and women in all group pairs except the early menopause pair (full reproductive age, P<0.0001; premenopause, P = 0.045; late menopause, P = 0.0012; Table S1).

No difference in the severity of liver necro-inflammation was observed between men and women in each group pair (Table S1).

Multivariate analysis in the whole male and female cohort, reported in Table 2, showed that sex (OR: 0.460, CI 0.236–0.896, P = 0.023), necro-inflammation (OR: 1.401, CI 1.239–1.584, P<0001), circulating estradiol levels (OR: 0.980, CI 0.962–0.999, P = 0.040), platelets count (OR: 0.974, 0.967–0.981, P<0.0001) and portal vein diameter (OR: 1.903, CI 0.539–2.354, P<0.0001) were independently related with severe fibrosis.

**Table 2:** Univariate and multivariate analysis for fibrosis in the whole cohort of patients with chronic hepatitis C.

	Univariate		Multivariate	
	OR (95% CI)	*P*	OR (95% CI)	*P*
**Sex** *	0.406 (0.254–0.649)	0.000	0.460 (0.236–0.896)	0.023
**Age (years)**	1.049 (1.027–1.072)	0.000	1.031 (1.000–1.062)	0.050
**Duration of HCV infection (years)**	1.064 (1.023–1.107)	0.002	0.989 (0.941–1.039)	0.654
**Necro-inflammation**	1.427 (1.312–1.553)	0.000	1.401 (1.239–1.584)	0.0001
**Steatosis (0 ** ***vs*** **. >20%)**	1.469 (1.062–2.032)	0.020	1.301 (0.840–2.016)	0.239
**Circulating Estradiol (pg/ml)**	0.982 (0.972–0.991)	0.000	0.980 (0.962–0.999)	0.040
**Blood Iron (ng/mL)**	1.010 (1.003–1.017)	0.006	1.018 (0.993–1.043)	0.170
**Ferritin (ng/mL)**	1.002 (1.000–1.003)	0.031	1.001 (0.995–1.006)	0.784
**ALT (IU/L)**	1.008 (1.005.011)	0.000	1.002 (0.998–1.006)	0.279
**GGT (IU/L)**	1.016 (1.010–1021)	0.000	1.003 (0.996–1.010)	0.387
**Platelet count (x10^3^/mm^3^)**	0.973 (0.967–0.979)	0.000	0.974 (0.967–0.981)	0.0001
**Portal vein diameter (mm)**	2.233 (1.901–2.622)	0.000	1.903 (0.539–2.354)	0.000

Only factors found to be significant by univariate analysis are reported.

* Male as reference. HCV, hepatitis C virus; BMI, body mass index; ALT, alanine aminotransferase; GGT, γ-glutamyl transpeptidase; OR, odds ratio; CI, confidence interval.

Results of multivariate analysis in the whole cohort, stratified in the four reproductive subgroups, are reported in Table 3. Male sex was independently associated with the severity of fibrosis in the premenopausal [male as reference: odds ratio (OR), 0.074; 95% confidence interval (CI), 0.007–0.834; P = 0.048] and early menopausal (OR, 0.037; 95% CI, 0.004–0.344; P = 0.004) group pairs, but not in the reproductive-aged or late menopausal group pairs (Table 3).

**Table 3:** Comparison of the baseline independent predictive factors for fibrosis in the four cohorts of patients with chronic hepatitis C.

	Reproductive-aged women/age-matched men (*n* = 123)				Premenopausal women/age-matched men (*n* = 38)				Early menopausal women/age-matched men (*n* = 50)				Late menopausal women/age-matched men (*n* = 144)			
	Univariate		Multivariate		Univariate		Multivariate		Univariate		Multivariate		Univariate		Multivariate	
	OR (95% CI)	*P*	OR (95% CI)	*P*	OR (95% CI)	*P*	OR (95% CI)	*P*	OR (95% CI)	*P*	OR (95% CI)	*P*	OR (95% CI)	*P*	OR (95% CI)	*P*
**Sex** *	0.231 (0.075–0.706)	0.010	0.869 (0.161–4.667)	0.870	0.071 (0.009–0.594)	0.015	0.074 (0.007–0.834)	**0.048**	0.112 (0.030–0.419)	0.001	0.037 (0.004–0.344)	**0.004**	0.794 (0.425–1.485)	0.471	§	
**Age (years)**	1.061 (0.998–1.127)	0.058	1.113 (0.931–1.332)	0.240	1.027 (0.676–1.560)	0.902	§		0.966 (0.857–1.089)	0.572	§		1.077 (0.983–1.181	0.110	§	
**Estimated duration of HCV infection (years)**	0.930 (0.809–1.069)	0.305	§		1.062 (0.913v1.236)	0.434	§		1.046 (0.959–1.140)	0.309	§		1.048 (0.992–1.106)	0.094	§	
**Mean BMI (kg/m^2^)**	0.954 (0.814–1.111)	0.544	§		1.032 (0.874–1.217)	0.711	§		1.119 (0.982–1.274)	0.090	0.997 (0.789–1.260)	0.981	0.974 (0.900–1.054)	0.512	§	
**Necro-inflammation**	1.370 (1.155–1.625)	0.000	1.448 (1.092–1.922)	**0.010**	1.433 (1.083–1.896)	0.012	1.501 (1.003–2.248)	**0.048**	1.556 (1.254–1.930)	0.000	1.727 (1.238–2.410)	**0.001**	1.407 (1.254–1.579)	0.000	1.566 (1.295–1.894)	**0.0001**
**Steatosis** **(0 ** ***vs*** **. >20%)**	2.782 (0.854–9.055)	0.089	1.874 (0.402–8.729)	0.424	2.038 (1.216–3.415)	0.007	5.242 (0.402–68.421)	0.206	1.455 (0.517–4.091)	0.478	§		1.177 (0.711–1.947)	0.527	§	
**Blood Iron** **(ng/mL)**	1.012 (1.001–1.024)	0.038	1.018 (0.993–1.043)	0.170	1.013 (0.987–1.038)	0.333	§		1.011 (0.998–1.025)	0.089	§		1.002 (0.987–1.017)	0.796	§	
**Ferritin** **(ng/mL)**	1.002 (1.000–1.005)	0.047	1.001 (0.995–1.006)	0.784	1.044 (0.995–1.013)	0.422	§		1.004 (1.000–1.009)	0.046	0.993 (0.978–1.008)	0.376	1.000 (0.997–1.002)	0.805	§	
**ALT (IU/L)**	1.007 (1.003–1.010)	0.001	1.002 (0.995–1.010)	0.549	1.010 (0.995–1.025)	0.178	§		1.008 (1.002–1.014)	0.006	0.999 (0.989–1.010)	0.880	1.010 (1.005–1.014)	0.000	1.006 (0.999–1.001)	0.102
**GGT (IU/L)**	1.021 (1.011–1031)	0.000	1.013 (1.000–1.026)	**0.045**	1.013 (0.998–1.028)	0.092	0.997 (0.979–1.015)	0.713	1.028 (1.010–1.046)	0.002	0.997 (0.982–1.012)	0.653	1.011 (1.004–1.018)	0.001	1.001 (0.993–1.009)	0.800
**Platelet count (×10^3^/mm^3^)**	0.976 (0.967–0.986)	0.000	0.982 (0.969–0.997)	**0.015**	0.972 (0.956–0.988)	0.001	0.973 (0.954–0.992)	**0.005**	0.971 (0.956–0.985)	0.000	0.975 (0.955–0.996)	**0.022**	0.972 (0.962–0.981)	0.000	0.967 (0.954–0.981)	**0.002**
**Portal vein diameter** **(mm)**	1.888 (1.416–2.516)	0.000	1.394 (0.5919–2.114)	0.118	3.822 (1.874–7.794)	0.000	4.611 (0.891–11.245)	**0.001**	2.111 (1.455–3.061)	0.000	2.316 (1.204–4.455)	**0.012**	5.960 (2.665–3.326)	0.000	4.729 (1.524–14.679)	**0.007**

Only factors found by univariate analysis to be significant in at least one of the four groups are reported.

* Male as reference; § Not included in multivariate model because not significant in univariate analysis.

HCV, hepatitis C virus; BMI, body mass index; ALT, alanine aminotransferase; GGT, γ-glutamyl transpeptidase; OR, odds ratio; CI, confidence interval.

### Hormone Assays

**Figure 2:**
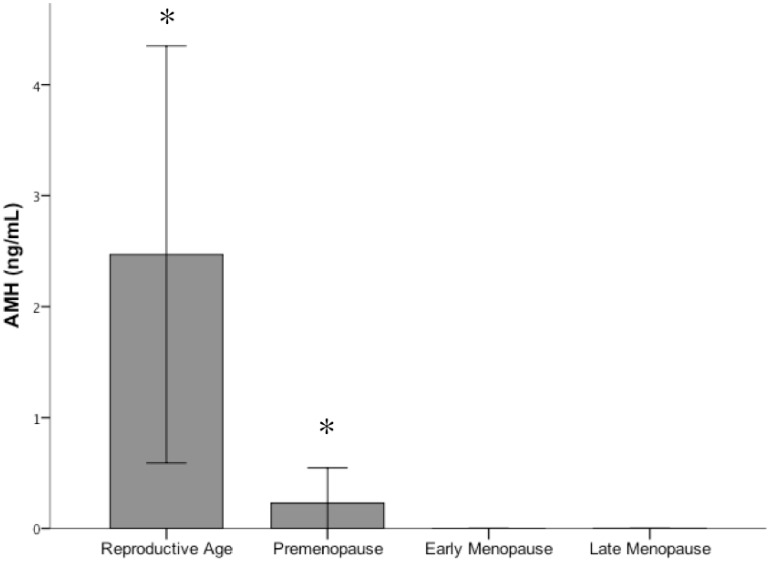
Mean serum levels of anti-müllerian hormone (AMH) in women divided according to reproductive phases. AMH levels were significantly lower in premenopausal women in comparison with women in full reproductive age (p<0.0001) and became undetectable in menopausal women (both early and late).

Serum concentrations of AMH in the four female groups are shown in Figure 2. Mean values were 2.4 (1.8) ng/mL in reproductive-aged women and 0.5 (0.3) ng/mL in premenopausal women (Mann–Whitney U-test, P<0.0001). AMH levels were undetectable in early and late menopausal women.

**Figure 3:**
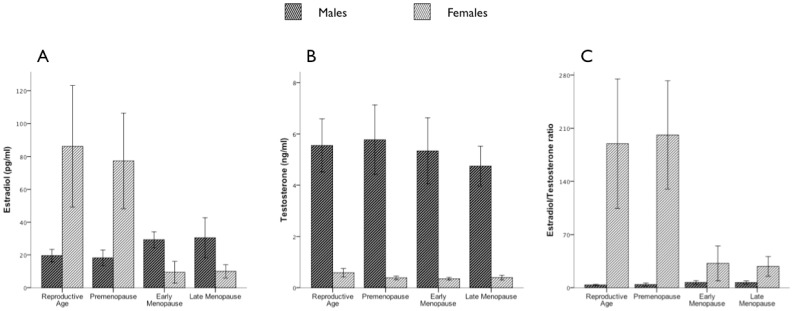
Estradiol and Testosterone serum levels and E2/T ratio in men and women divided according to women’s reproductive phases as described in Methods. Values (reported as mean±SD) were compared by Mann-Whitney test. The levels of significance are reported in the text.

Serum concentrations of estradiol and testosterone in the four group pairs are shown in Figures 3A and 3B, respectively, and the estradiol/testosterone ratio [E2/T; estradiol (pg/mL)/testosterone (ng/mL)] is reported in Figure 3C. Estradiol concentrations changed significantly in both men and women with age, with a marked difference observed in both sexes at the age corresponding to female menopausal onset. In particular, estradiol levels were significantly higher in reproductive-aged and premenopausal women than in early (reproductive age: P<0.0001; premenopausal: P<0.0001) and late menopausal women (reproductive age: P<0.0001; premenopausal: P<0.0001), whereas males exhibited a significant change in the opposite direction at corresponding ages (reproductive vs. early menopause: P = 0.003, vs. late menopause: P = 0.05; premenopausal groups vs. early: P = 0.001, vs. late P = 0.005). Testosterone levels changed significantly only in women, who showed significantly higher levels in the full reproductive age group than in the other groups (reproductive vs. premenopausal P = 0.44; vs. early menopausal P = 0.006; vs. late menopausal P = 0.013). Although a decline in testosterone levels was observed in the two older groups of men, this difference was not significant. The E2/T ratio changed significantly with age in both men and women. In women, the E2/T ratio decreased markedly in early (vs. reproductive age: P = 0.002 and vs. premenopausal: P<0.0001) and late menopause (vs. reproductive age: P = 0.001 and vs. premenopausal: P<0.0001). The opposite change occurred in men (reproductive age group vs. early menopause group: P = 0.002 and vs. late: P = 0.001) (Figure 3C).

## Discussion

In this cross-sectional study comparing a large cohort of women with CHC who were grouped, according to reproductive status, with age-matched men with CHC, we found that hormonal phases were differently associated with the histological severity of liver disease.

A few clinical studies [8], [9] have found that estrogens exert a beneficial effect on liver disease by slowing the progression of fibrosis; however, this effect has previously been evaluated in terms of lifetime exposure, rather than according to different reproductive stages. To overcome this limitation, we divided a cohort of 355 women with CHC into four groups according to reproductive status. The allocation of patients to these groups was substantiated using AMH levels, because this glycoprotein is a more accurate indicator of ovarian reserve than is age or other conventional serum marker (follicle stimulating hormone, estradiol, inhibin B) [14]–[16]. The AMH levels obtained in our study were in agreement with those reported for reproductive age, premenopause, and established menopause [17], [18], and thus validated the composition of the four groups.

The results of our study support the concept that the progression of fibrosis in women is a discontinuous process: very slow during reproductive age and accelerating rapidly after menopause. We found that disease severity at the time of liver biopsy, as measured by the severity of liver necro-inflammation and fibrosis, was very low in women of reproductive age and in premenopausal ones while it became higher among early menopausal women; the severity of fibrosis was found to be even higher among late menopausal women. Further support for the protective role of estrogens toward development of fibrosis comes from the multivariate analysis of risk factors for severe fibrosis: neither estimated duration of HCV infection nor the age of acquisition of HCV infection was independently associated with fibrosis while levels of circulating estradiol was.

The impact of hormonal exposure on fibrosis became even more complex when the female groups were compared with their age-matched male counterparts. Although the estimated duration of HCV infection was similar in all group pairs and age was equivalent by definition, women had less severe fibrosis than men in all but the late menopausal group pair. Female sex appeared to be independently associated with a lower prevalence of severe fibrosis in the premenopausal and early menopausal group pairs [19].

These findings may be explained by the observed changes in hormone levels according to reproductive status. The high estradiol levels observed in the reproductive-aged groups (full reproductive, premenopause) likely provided protection against the development of severe liver injury. However, an independent correlation between fibrosis and sex was found only in the premenopausal and early menopausal group pairs, and not in the fully reproductive group pair. This finding may be due to the short duration of infection and to the very mild stage of disease present in both sexes in the reproductive-aged group pair.

In contrast, menopausal women exhibited greater disease severity than those in the reproductive-aged and premenopausal group pairs, probably due to the decline in estradiol levels that occurs rapidly with menopause development. We have already shown that a marked up-regulation of inflammatory cytokines, especially interleukin-6 (IL-6), occurs in close temporal relationship with the onset of menopause [11], [12], when for the first time in a woman’s life E2/T ratio dramatically decreases: this is associated with a sharp increase in necro-inflammatory activity and is eventually followed (as the change in fibrosis score between early and menopausal women shows) by a significant increase in fibrosis in late menopausal women. As the late menopausal male-female group comparison indicates, at this stage women lose the advantage that they had in earlier reproductive stages: fibrosis and percentage of cirrhosis are no longer significantly different from males and only subtle indicators of disease progression (like portal vein diameter and platelets count) are still better in females. A further element that may play a relevant role in reducing differences in disease severity between genders in the late groups is the significant increase of estradiol levels and E2/T ratio that was observed in males of the early and late menopausal groups. This increase did not result in a change in necro-inflammation, but was associated with a significant reduction in fibrosis severity in men in group 4 (group 4 vs. group 3, P = 0.010). This is in agreement with the recently reported positive correlation between testosterone levels and increased risk of both advanced hepatic fibrosis and advanced hepatic inflammatory activity in HCV-infected men [20]: although this study evaluated only absolute Testosterone levels and not Estradiol or the E2/T ratio, it is relevant that higher unopposed levels of Testosterone related with higher severity of disease. In our cohort, the late inversion of the E2/T ratio in males coincided with the first and only improvement in fibrosis score in males.

These findings not only reinforce the view that the lengthy period of exposure to estrogens in women plays a favorable role in the progression of chronic liver disease in comparison with men, but also suggest that this protective effect is reversible and strictly dependent on estrogen levels. When the process of hormonal aging leads to an inversion in the E2/T balance characteristic of full reproductive age, women lose the favorable estrogen effect and show patterns similar to those of men. Accordingly, male sex is constantly and independently associated with a higher degree of fibrosis in cohorts whose mean age coincides with the full reproductive stage (i.e., <50 years of age) [5], [6]. In contrast, cohorts with higher mean ages (>50 years; a surrogate indicator of menopause in women) show similar patterns of fibrosis progression in men and women, and male sex is no longer a predictor of severe fibrosis progression [6], [21], [22].

Although our study was not designed to clarify the pathogenesis of the association between reproductive phase and the severity of fibrosis in women, previous studies have provided several hypotheses. Many experimental data have shown that estradiol inhibits transforming growth factor (TGF)-β1 expression and hepatic stellate cell (HSC) activation, thereby suppressing the induction of hepatic fibrosis [8], [23], [24]. Estradiol is also known to induce the down-regulation of tumor necrosis factor (TNF) alpha, IL-6, and IL-1β [25]–[27], mediators contributing to hepatic necro-inflammation and the activation of HSCs. The favorable role of estrogens in men has also been suggested by two case reports, in which different pathological conditions required prolonged estrogen treatment in two young men, one with hepatitis C [28] and the other with nonalcoholic steatohepatitis (NASH) [29]. In the first case, the addition of estradiol reduced disease activity and maintained viral loads at lower levels than observed before treatment [28]. In the second case, it reversed hepatic steatosis and insulin resistance [28].

In this study of a cohort of women with CHC classified by reproductive phase and compared with age-matched men with CHC, we found that the severity of fibrosis in women is strictly related with Estradiol levels and E2/T ratio. Progression of fibrosis follows the sharp rise of necro-inflammatory activity occurring in coincidence with estrogen deprivation in early menopause [11]. This finding explains the discontinuous rate of fibrosis progression in women and the more linear rate observed in men, in whom the modification of sex hormone balance determined by aging-associated Estradiol increase occurs very late in life and thus may have only a limited beneficial effect on hepatic condition.

### Limitations

The main limitation of this study lies in its cross-sectional design, which prevented the evaluation of fibrosis progression in individual patients according to baseline reproductive status and reproductive transitions. A further methodological question is the potentially limited external validity of the results for different populations and settings, because our study included a cohort of Italian subjects enrolled at a tertiary care center.

## Supporting Information

Table S1 Comparison between female and age-matched male patients with chronic hepatitis.
(DOC)

Table S2 Comparison between female and age-matched male patients with chronic hepatitis.
(DOC)

Table S3 Comparison between the four groups of female patients with chronic hepatitis C.
(DOC)

Table S4 Comparison between the four groups of female patients with chronic hepatitis C.
(DOC)
