# Supplementary material for: Reproductive Status Is Associated with the Severity of Fibrosis in Women with Hepatitis C
Source: PLoS One. 2012 Sep 10;7(9):e44624. doi: 10.1371/journal.pone.0044624 (PMC3438179; doi:10.1371/journal.pone.0044624)
Supplement: Table S1 — Comparison between female and age-matched male patients with chronic hepatitis. (DOC) [file pone.0044624.s001.doc]

Supporting information.

**Table S1 -** Comparison between female and age-matched male patients with chronic hepatitis.

|  | **Women of reproductive age (123)** | **Age-matched men (123)** | **P** | **Pre-menopausal women (38)** | **Age-matched men (38)** | **P** | **Early menopausal women (50)** | **Age-matched men (50)** | **P** | **Late menopausal**  **Women (144)** | **Age-matched men (144)** | **P** |
| --- | --- | --- | --- | --- | --- | --- | --- | --- | --- | --- | --- | --- |
| **Mean age, years (SD, range)** | 36.7 (6.9, 18–45) | 36.6 (4.7, 20–45) | NS | 47.6 (1.8, 46–50) | 47.5 (1.4, 45–50) | NS | 53.8 (3.6, 47–60) | 54.0 (4.6, 48–60) | NS | 62.3 (3.2; 55–73) | 62.1 (3.8; 53–74) | NS |
| **Mean duration of HCV infection (years)(SD)** | 11.0 (4.1) | 12.1 (4.4) | NS | 12.1 (3.2) | 12.39 (5.2) | NS | 14.4 (5.9) | 14.7 (5.1) | NS | 14.9 (3.8) | 15.2 (6.0) | NS |
| **Mean BMI (SD)** | 23.8 (3.8) | 26.8 (3.6) | 0.0001 | 24.9 (3.8) | 26.7 (3.9 | 0.045 | 25.0 (4.0) | 26.2 (3.4) | NS | 25.2 (4.2) | 26.4 (3.8) | 0.001 |
| **Mean grade (SD)** | 3.4 (2.0) | 3.8 (2.3) | NS | 3.11 (2.5) | 4.10 (2.2) | NS | 4.4 (2.7) | 4.0 (3.0) | NS | 3.4 (3.0) | 3.7 (2.6) | NS |
| **Mean stage (SD)** | 1.4 (1.0) | 1.8 (1.1) | 0.001 | 1.58 (0.2) | 2.6 (.2) | 0.001 | 1.9 (0.9) | 3.1 (1.6) | 0.001 | 2.3 (1.1) | 2.5 (1.2) | NS |
| **Cirrhosis at enrollment, n (%)** | 1 (0.8) | 3 (2.4) | NS | 0 | 8 (21.1) | 0.003 | 3 (6.0) | 13 (26.0) | 0.003 | 17 (11.8) | 26 (18.0 | NS |
| **Mean portal vein diameter, (mm) (SD)** | 10.1 (1.0) | 10.6 (1.3) | 0.002 | 10.3 (0.8) | 11.2 (1.9) | 0.005 | 10.2 (1.5) | 11.6 (1.8) | 0.0001 | 10.6 (1.6) | 11.3 (1.6 | 0.001 |
| **Mean GGT (IU/L)(SD)** | 29 (22) | 50 (40) | <0.0001 | 28 (13) | 70 (74) | 0.0001 | 33 (22) | 61 (69) | 0.002 | 42 (30) | 56 (65 | NS |
| **Mean ALT (IU/L)(SD)** | 66 (60) | 99 (78) | <0.0001 | 54 (32) | 82 (44) | 0.002 | 81 (73) | 108 (97) | NS | 75 (65) | 89 (71) | NS |
| **Mean Viral load (IU/mL)(SD)** | 1.118.390 (196.28E6) | 2.117.473 (429.076E6) | <0.0001 | 1.092.013 (1.785E6) | 1.534.580 (2.346E6) | NS | 1.495.754 (2.132E6) | 1.176.966 (1.656E6) | NS | 1.185.729 (2.376E6) | 1.277.404 (2.230E6) | NS |
| **Mean Cholesterol (mg/dL)(SD)** | 166 (35) | 157 (40) | NS | 175 (37) | 161 (45) | NS | 184.8 (46.9) | 152 (31) | 0.003 | 183 (3) | 176.(36) | NS |
| **Mean HDL cholesterol (mg/dL)(SD)** | 60 (13) | 50 (14) | 0.017 | 76 (17) | 48 (6) | 0.038 | 75 (26) | 52 (7) | 0.026 | 63.1 (23) | 57 (26) | NS |
| **Mean Triglycerides (mg/dL)(SD)** | 92 (46) | 104 (58) | NS | 86.2 (39) | 126 (86) | NS | 76 (45) | 96 (27) | 0.01 | 81.5 (23) | 104 (41) | 0.038 |
| **Mean Ferritin (ng/mL)(SD)** | 72 (85) | 281 (72) | <0.0001 | 80 (119) | 301 (386) | 0.002 | 100 (74) | 382 (251) | 0.0001 | 227 (187) | 407 (377) | 0.011 |
| **Mean Blood glucose (mg/dL)(SD)** | 86 (9) | 91 (16) | NS | 90 (10) | 92 (8) | NS | 94 (18) | 97.8 (13) | 0.045 | 94 (15) | 97.6 (16) | NS |
| **Mean Platelet count(x10^3^/m^3^)** | 234 (59) | 198 (56) | <0.0001 | 223 (61) | 180 (63 | 0.003 | 194 (72) | 157 (46) | 0.001 | 184 (61) | 161 (55) | 0.003 |

HCV, hepatitis C virus; BMI, body mass index; GGT, γ-glutamyl transpeptidase; ALT, alanine aminotransferase; HDL, high-density lipoprotein.
