# Supplementary material for: Reproductive Status Is Associated with the Severity of Fibrosis in Women with Hepatitis C
Source: PLoS One. 2012 Sep 10;7(9):e44624. doi: 10.1371/journal.pone.0044624 (PMC3438179; doi:10.1371/journal.pone.0044624)
Supplement: Table S2 — Comparison between female and age-matched male patients with chronic hepatitis. (DOC) [file pone.0044624.s002.doc]

Supporting information.

**Table S2 -** Comparison between female and age-matched male patients with chronic hepatitis.

|  |  | **Women of reproductive age (123)** | **Age-matched men (123)** | **P** | **Pre-menopausal women (38)** | **Age-matched men (38)** | **P** | **Early menopausal women (50)** | **Age-matched men (50)** | **P** | **Late menopausal**  **Women (144)** | **Age-matched men (144)** | **P** |
| --- | --- | --- | --- | --- | --- | --- | --- | --- | --- | --- | --- | --- | --- |
| **HCV Genotype, n (%)** | 1 | 66 (55.3) | 57 (46.7) | NS | 19 (50.0) | 22 (57.9) | NS | 23 (46.0) | 30 (60.0) | NS | 90 (62.5) | 77 (53.5) | NS |
|  | 2 | 30 (24.4) | 16 (13.1) | 0.034 | 14 (36.8) | 6 (15.8) | NS | 18 (36.0) | 14 (28.0) | NS | 51 (35.4) | 62 (43.1) | NS |
|  | 3 | 19 (15.4) | 39 (32.0) | 0.004 | 4 (10.5) | 8 (21.1) | NS | 6 (12.0) | 4 (8.9) | NS | 0 (0) | 2 (1.4) | NS |
|  | 4 | 6 (4.9) | 10 (8.2) | NS | 1 (2.6) | 2 (5.3) | NS | 3 (6.0) | 2 (4.0) | NS | 3 (2.1) | 3 (2.1) | NS |
| **Source of infection, n (%)** | **Community-acquired** | 83 (68.0) | 65 (53.2) | 0.027 | 31 (81.6) | 28 (63.6) | NS | 37 (74.0) | 40 (80.0) | NS | 98 (68.8) | 16 (81.4) | 0.027 |
|  | **Parenteral exposure** | 31 (25.4) | 18 (14.8) | NS | 7 (18.4) | 5 (13.2) | NS | 13 (26.0) | 7 (14.0) | NS | 46 (32.2) | 23 (16.2) | 0.002 |
|  | **Drug addiction** | 9 (7.4) | 38 (31.2) | <0.0001 | 0 (0) | 5 (13.2) | NS | 0 (0) | 3 (6.0) | NS | 0 (0) | 2 (1.4) | NS |
| **Histology, n (%)** | **Steatosis Absent** | 86 (70.8) | 71 (58.4) | NS | 21 (56.0) | 22 (58.6) | NS | 30 (61.2) | 33 (67.4) | NS | 83 (58.4) | 96 (67.6) | NS |
|  | **Steatosis <10%** | 18 (14.8) | 23 (19.0) | NS | 5 (13.4) | 5 (13.4) | NS | 8 (16.4) | 6 (12.2) | NS | 27 (19.0) | 26 (18.4) | NS |
|  | **Steatosis ≥10% to <20%** | 11 (9.0) | 11 (9.0) | NS | 8 (21.4) | 8 (21.4) | NS | 6 (12.2) | 8 (16.4) | NS | 18 (12.6) | 16 (11.2) | NS |
|  | **Steatosis ≥20% to <30%** | 2 (1.6) | 8 (6.6) | NS | 0 (0) | 2 (2.7) | NS | 3 (6.2) | 1 (2.0) | NS | 5 (3.6) | 2 (1.4) | NS |
|  | **Steatosis >30%** | 4 (3.2) | 9 (7.4) | NS | 3 (8.0) | 1 (2.6) | NS | 2 (4.0) | 1 (2.0) | NS | 8 (5.6) | 3 (2.2) | NS |
| **Grading, n (%)** | **0**–**6** | 109 (93.6) | 102 (87.6) | NS | 31 (87.4) | 30 (84.6) | NS | 38 (84.4) | 35 (77.8) | NS | 120 (89.8 | 109 (81.6) | NS |
|  | **7**–**12** | 7 (6.0) | 15 (12.8) | NS | 5 (14.0) | 5 (7) | NS | 7 (15.6) | 9 (20) | NS | 12 (9.0) | 22 (16.4) | NS |
|  | **13**–**18** | - | - | - | - | - | - | 0 (0) | 1 (2.2) | NS | 4 (3.0) | 0 (0) | NS |
| **Staging, n (%)** | **0**–**2** | 103 (88.4) | 93 (79.8) | NS | 30 (84.6) | 21 (59.2) | 0.050 | 34 (74.8) | 20 (44) | 0.009 | 88 (65.4) | 71 (52.8) | NS |
|  | **3**–**4** | 11 (9.4) | 17 (14.6) | NS | 5 (14.0) | 11 (31.0) | NS | 11 (24.2) | 14 (30.8) | NS | 41 (30.4) | 52 (38.4) | NS |
|  | **5**–**6** | 2 (1.8) | 7 (6.0) | NS | 1 (2.8) | 3 (8.4) | NS | 1 (2.2) | 11 (24.2) | 0.006 | 7 (5.2) | 10 (7.4) | NS |

HCV, hepatitis C virus; BMI, body mass index; GGT, γ-glutamyl transpeptidase; ALT, alanine aminotransferase; HDL, high-density lipoprotein.
