# Supplementary material for: Reproductive Status Is Associated with the Severity of Fibrosis in Women with Hepatitis C
Source: PLoS One. 2012 Sep 10;7(9):e44624. doi: 10.1371/journal.pone.0044624 (PMC3438179; doi:10.1371/journal.pone.0044624)
Supplement: Table S3 — Comparison between the four groups of female patients with chronic hepatitis C. (DOC) [file pone.0044624.s003.doc]

Supporting information.

**Table S3 -** Comparison between the four groups of female patients with chronic hepatitis C. Continuous data were analyzed by Mann-Whitney U test and categorical data by chi square test. P was indicated when significant or borderline significant.

|  | **Group 1- Women of reproductive age (123)** | **Group 2-Premenopausal women (38)** | **Group 3- Early menopausal women (50)** | **Group 4- Late menopausal women (144)** | **G1 vs. G2 p** | **G1 vs. G3 p** | **G1 vs. G4 p** | **G2 vs. G3 p** | **G2 vs. G4 p** | **G3 vs. G4 p** |
| --- | --- | --- | --- | --- | --- | --- | --- | --- | --- | --- |
| **Mean age, years (SD, range)** | 36.7 (6.9, 18-45) | 47.6 (1.8, 46-50) | 53.8 (3.6, 47-60) | 62.3 (3.2, 55-73) | <0.0001 | <0.0001 | <0.0001 | 0.0001 | <0.0001 | <0.0001 |
| **Mean duration of HCV infection (years)(SD)** | 11.0 (4.1) | 12.1 (3.2) | 14.4 (5.9) | 14.9 (3.8) | 0.067 | <0.0001 | <0.0001 | 0.017 | <0.0001 | NS |
| **Mean BMI (SD)** | 23.8 (3.8) | 24.9 (3.8) | 25.0 (4.0) | 25.2 (4.2) | NS | 0.039 | 0.004 | NS | NS | NS |
| **Mean grade (SD)** | 3.4 (2.0) | 3.1 (2.5) | 4.4 (2.7) | 3.4 (3.0) | NS | 0.020 | NS | 0.014 | NS | 0.004 |
| **Mean stage (SD)** | 1.4 (1.0) | 1.58 (0.2) | 1.9 (0.9) | 2.3 (1.1) | 0.062 | <0.0001 | <0.0001 | 0.042 | <0.0001 | 0.052 |
| **Cirrhosis at enrollment, n (%)** | 1 (0.8) | 0 | 3 (6.0) | 17 (11.8) | NS | 0.039 | 0.004 | NS | 0.026 | NS |
| **Mean portal vein diameter (mm) (SD)** | 10.1 (1.0) | 10.3 (0.8) | 10.2 (1.5) | 10.6 (1.6) | NS | NS | 0.001 | NS | NS | 0.094 |
| **Mean GGT (IU/L)(SD)** | 29 (22) | 28 (13) | 33 (22) | 42 (30) | NS | NS | <0.0001 | NS | 0.009 | 0.075 |
| **Mean ALT (IU/L)(SD)** | 66 (60) | 54 (32) | 81 (73) | 75 (65) | NS | NS | NS | NS | NS | NS |
| **Mean Viral load (IU/mL)(SD)** | 1.118.390 (196.28E6) | 1.092.013 (1.785 E6) | 1.495.754 (2.132E6) | 1.185.729 (2.376E6) | NS | NS | NS | NS | NS | NS |
| **Mean Cholesterol (mg/dL)(SD)** | 166 (35) | 175 (37) | 184.8 (46.9) | 183 (3) | NS | 0.013 | 0.008 | NS | NS | NS |
| **Mean HDL cholesterol (mg/dL)(SD)** | 60 (13) | 76 (17) | 75 (26) | 63.1 (23) | 0.043 | 0.046 | NS | NS | NS | NS |
| **Mean Triglycerides (mg/dL)(SD)** | 92 (46) | 86.2 (39) | 76 (45) | 81.5 (23) | NS | NS | NS | NS | NS | 0.023 |
| **Mean Ferritin (ng/mL)(SD)** | 72 (85) | 80 (119) | 100 (74) | 227 (187) | NS | 0.005 | <0.0001 | NS | 0.001 | 0.001 |
| **Mean Blood glucose (mg/dL)(SD)** | 86 (9) | 90 (10) | 94 (18) | 94 (15) | NS | 0.011 | <0.0001 | NS | 0.054 | NS |
| **Mean Platelet count (x10^3^/mm^3^) (SD)** | 234 (59) | 223 (61) | 194 (72) | 184 (61) | NS | <0.0001 | <0.0001 | 0.029 | <0.0001 | NS |

HCV, hepatitis C virus; BMI, body mass index; GGT, γ-glutamyl transpeptidase; ALT, alanine aminotransferase; HDL, high-density lipoprotein.
