# Supplementary material for: Reproductive Status Is Associated with the Severity of Fibrosis in Women with Hepatitis C
Source: PLoS One. 2012 Sep 10;7(9):e44624. doi: 10.1371/journal.pone.0044624 (PMC3438179; doi:10.1371/journal.pone.0044624)
Supplement: Table S4 — Comparison between the four groups of female patients with chronic hepatitis C. (DOC) [file pone.0044624.s004.doc]

Supporting information.

**Table S4 -** Comparison between the four groups of female patients with chronic hepatitis C.

|  |  | **Group 1 - Women of reproductive age (123)** | **Group 2-Premenopausal women (38)** | **Group 3 - Early menopausal women (50)** | **Group 4 - Late menopausal women (144)** | **G1 vs. G2 p** | **G1 vs. G3 p** | **G1 vs. G4 p** | **G2 vs. G3 p** | **G2 vs. G4 p** | **G3 vs. G4 p** |
| --- | --- | --- | --- | --- | --- | --- | --- | --- | --- | --- | --- |
| **HCV Genotype, n (%)** | **1** | 66 (55.3) | 19 (50.0) | 23 (46.0) | 90 (62.5) | NS | NS | NS | NS | NS | 0.041 |
|  | **2** | 30 (24.4) | 14 (36.8) | 18 (36.0) | 51 (35.4) | NS | NS | 0.050 | NS | NS | NS |
|  | **3** | 19 (15.4) | 4 (10.5) | 6 (12.0) | 0 | NS | NS | <0.0001 | NS | <0.0001 | <0.0001 |
|  | **4** | 6 (4.9) | 1 (2.6) | 3 (6.0) | 3 (2.1) | NS | NS | NS | NS | NS | NS |
| **Source of infection, n (%)** | **Community-acquired** | 83 (68.0) | 31 (81.6) | 37 (74.0) | 98 (68.8) | NS | NS | NS | NS | NS | NS |
|  | **Parenteral exposure** | 31 (25.4) | 7 (18.4) | 13 (26.0) | 46 (32.2) | NS | NS | NS | NS | NS | NS |
|  | **Drug addiction** | 9 (7.4) | 0 (0) | 0 (0) | 0 (0) | NS | 0.049 | 0.001 | - | - | - |
| **Histology, n (%)** | **Steatosis Absent** | 86 (70.8) | 21 (56.0) | 30 (61.2) | 83 (58.4) | NS | NS | 0.038 | NS | NS | NS |
|  | **Steatosis <10%** | 18 (14.8) | 5 (13.4) | 8 (16.4) | 27 (19.0) | NS | NS | NS | NS | NS | NS |
|  | **Steatosis ≥10% to <20%** | 11 (9.0) | 8 (21.4) | 6 (12.2) | 18 (12.6) | 0.043 | NS | NS | NS | NS | NS |
|  | **Steatosis ≥20% to <30%** | 2 (1.6) | 0 (0) | 3 (6.2) | 5 (3.6) | NS | NS | NS | NS | NS | NS |
|  | **Steatosis ≥30%** | 4 (3.2) | 3 (8.0) | 2 (4.0) | 8 (5.6) | NS | NS | NS | NS | NS | NS |
| **Grading, n (%)** | **0**–**6** | 109 (93.6) | 31 (87.4) | 38 (84.4) | 120 (89.8) | NS | 0.035 | NS | NS | NS | NS |
|  | **7**–**12** | 7 (6.0) | 5 (14.0) | 7 (15.6) | 12 (9.0) | NS | NS | NS | NS | NS | NS |
|  | **13**–**18** | - | - | 0 (0) | 4 (3.0) | - | - | - | - | - | NS |
| **Staging, n (%)** | **0**–**2** | 103 (88.4) | 30 (84.6) | 34 (74.8) | 88 (65.4) | NS | 0.020 | <0.0001 | NS | 0.040 | NS |
|  | **3**–**4** | 11 (9.4) | 5 (14.0) | 11 (24.2) | 41 (30.4) | NS | 0.019 | <0.0001 | NS | NS | NS |
|  | **5**–**6** | 2 (1.8) | 1 (2.8) | 1 (2.2) | 7 (5.2) | NS | NS | NS | NS | NS | NS |

HCV, hepatitis C virus; BMI, body mass index; GGT, γ-glutamyl transpeptidase; ALT, alanine aminotransferase; HDL, high-density lipoprotein.
